# Social media use in healthcare: A systematic review of effects on patients and on their relationship with healthcare professionals

**DOI:** 10.1186/s12913-016-1691-0

**Published:** 2016-08-26

**Authors:** Edin Smailhodzic, Wyanda Hooijsma, Albert Boonstra, David J. Langley

**Affiliations:** 1 Faculty of Economics and Business, University of Groningen, Groningen, The Netherlands; 2 TNO, Netherlands Organization for Applied Scientific Research, Groningen, The Netherlands

**Keywords:** Social media, Health, Patients, Healthcare professionals

## Abstract

**Background:**

Since the emergence of social media in 2004, a growing percentage of patients use this technology for health related reasons. To reflect on the alleged beneficial and potentially harmful effects of social media use by patients, the aim of this paper is to provide an overview of the extant literature on the effects of social media use for health related reasons on patients and their relationship with healthcare professionals.

**Methods:**

We conducted a systematic literature review on empirical research regarding the effects of social media use by patients for health related reasons. The papers we included met the following selection criteria: (1) published in a peer-reviewed journal, (2) written in English, (3) full text available to the researcher, (4) contain primary empirical data, (5) the users of social media are patients, (6) the effects of patients using social media are clearly stated, (7) satisfy established quality criteria.

**Results:**

Initially, a total of 1,743 articles were identified from which 22 were included in the study. From these articles six categories of patients’ use of social media were identified, namely: emotional, information, esteem, network support, social comparison and emotional expression. The types of use were found to lead to seven identified types of effects on patients, namely improved self-management and control, enhanced psychological well-being, and enhanced subjective well-being, diminished subjective well-being, addiction to social media, loss of privacy, and being targeted for promotion. Social media use by patients was found to affect the healthcare professional and patient relationship, by leading to more equal communication between the patient and healthcare professional, increased switching of doctors, harmonious relationships, and suboptimal interaction between the patient and healthcare professional.

**Conclusions:**

Our review provides insights into the emerging utilization of social media in healthcare. In particular, it identifies types of use by patients as well as the effects of such use, which may differ between patients and doctors. Accordingly, our results framework and propositions can serve to guide future research, and they also have practical implications for healthcare providers and policy makers.

**Electronic supplementary material:**

The online version of this article (doi:10.1186/s12913-016-1691-0) contains supplementary material, which is available to authorized users.

## Background

Previous studies on social media use in healthcare identified different effects of social media use by patients for health related reasons within the healthcare system. Social media can serve as an aid to patients. For example, it fosters their autonomy by complementing the information provided by healthcare professionals [1] and by providing psychosocial support [2]. Social media use by patients can also be an aid to healthcare professionals by providing a tool to strengthen the organization’s market position [3, 4] and stimulating conversation for brand building and improved service delivery [4, 5]. In fact, social media may have effects on both patients, and on the wider healthcare system [6]. In particular, it allows patients to receive support [1], and to complement offline information [2], which may lead to enhancing the empowerment of patients [6]. However, social media use by patients does not only provide beneficial effects. It may also constitute a challenge within the healthcare system to both patients and healthcare professionals. Since everybody with access to social media can post “advice” on how to deal with a certain health condition, it is important to create reliable online communication channels to prevent health problems being exacerbated [7]. For example, one misguided idea on Twitter urged Nigerians to drink excessive amounts of salt water to combat Ebola. However, this may have led to two deaths and more than 12 admissions to hospital [7]. Thus, many healthcare professionals fear that social media use by patients for health related purposes often spreads misinformation among patients [1].

Use of social media by patients for health related reasons provides different effects, which can result in both benefits and challenges. It is important to identify these effects of social media for the healthcare system, as “a growing percentage of patients use social media for health-related reasons, so health professionals will have to reflect on the alleged beneficial effects and the potential harmful effects of social media use by patients in healthcare” [8]. Hence, the review of these effects will contribute to a better understanding of potential benefits and challenges for both patients and healthcare professionals, but also other healthcare actors such as policy makers.

Therefore, this paper provides a systematic literature review of empirical studies on the effects of social media use by patients for health related reasons on patients and on their relationships with healthcare professionals. To our knowledge no other systematic research on this topic has been performed to date. Such review also provides the opportunity to extract general findings from the studies. Subsequently, healthcare professionals can learn from these findings about the effects of social media use by patients and share this knowledge with other patients and use it to their own advantage. We aim to answer the following question:

*According to recent empirical research, what are the effects of social media use by patients for health related reasons on patients and on their relationships with healthcare professionals?*

To answer this question, the paper will address the following: (1) the types of social media use by patients (2) the identified effects of social media use by patient on patients (3) the identified effects on the relationship between patients and their healthcare professionals and (4) the relationship between the effects on patients and healthcare professionals. By addressing the issue (4), we attempt to bring together our findings from the issues (2) and (3) and explore linking mechanisms between the effects patients experience and their subsequent link to the effects they experience in relationship with the healthcare professionals.

### Study aim and terminology

The aim of this paper is to gain insights in the benefits and challenges of the effects of social media use by patients within the healthcare system and especially the effects on patients and on their relationships with healthcare professionals. The effects we focus on in this paper can be both causal and reciprocal, but always start with the use of social media by patients.

Despite the popularity of social media, there is a confusion about what is exactly meant by the term social media. Therefore, in this paper we use the definition provided in the highly cited paper by Kaplan and Haenlein [9]. They describe social media as “a group of Internet-based applications that build on the ideological and technological foundations of Web 2.0, and that allow the creation and exchange of User Generated Content”. The internet-based applications refer to the different categories of social media, which are blogs, content communities, social networking sites, collaborative projects, virtual game worlds and virtual social worlds. These types of social media are accessible to users to utilize for, among other things, health related reasons.

The term “users of social media in healthcare” in this paper refer to the patients and their family members. Patients are treated as any person who self-proclaims to be suffering from a certain condition, whether officially diagnosed by a healthcare professional or not. We define healthcare professionals as those who study, advise on or provide preventive, curative, rehabilitative and promotional health services based on an extensive body of theoretical and factual knowledge in diagnosis and treatment of conditions and other health problems [10].

## Methods

In order to provide an overview of the different effects of social media use by patients for health related reasons on patients and on their relationships with healthcare professionals, we conducted systematic literature review.

To identify the articles, we employed a search strategy consisting of three terms as follows

a) “social media” or blog* or “content communit*” or “social networking site*” or “online social network*” or “virtual world*” or “online communit*” or “online forum*” or Facebook or Twitter or Wikipedia or IMVU or “second life” or YouTube b) “Patient*” and c) “health* provider*” or “health* professional*” or “physician*” or “doctor*” or “hospital*”. The full search string is also included in the Appendix A (see Additional file 1). Additionally, as suggested by the referees of this paper, we also used the term “client*” instead of “patient*”, together with the other two original categories of terms.

To perform this literature review, we followed the guidelines on conducting a systematic literature review as prescribed by the Preferred Reporting Items for Systematic Literature Reviews and Meta-Analyses (PRISMA) [11].

To conduct the search, we chose relevant databases of Web of Science and EBSCOhost COMPLETE. By focusing on EBSCOhostCOMPLETE, we made sure that the healthcare databases are included such as “PsycINFO”, “CINAHL” and “MEDLINE”. We also included the databases such “Business source premier” to include findings with a business perspective. Search options were slightly different for each database. For EBSCO the irrelevant databases were excluded first and no specific search field was selected for one of the three terms. The list of databases is presented in the Appendix B (See Additional file 2). Additionally, the option to search only in scholarly (peer reviewed) journals was used and the publication dates were selected to be after 2004. In the year 2004 the term Web 2.0 was used for the first time, which marks the start of the social media era [9]. On the other hand, we selected topic for all three terms in the Web of Science, which included the titles, abstracts, author keywords, and keywords plus fields of the articles.

### Selection criteria

For an article to be included in the study it had to meet several selection criteria as follows: (1) published in a peer-reviewed journal, (2) written in English, (3) full text available to the researcher, (4) contain primary empirical data, (5) the users of social media are patients, (6) the effects of patients using social media are clearly stated, (7) satisfy established quality criteria. The articles were assessed on their quality by using the standard quality assessment criteria as identified by [12].

Prior to final screening and selection of the papers, first and second author agreed to independently read 100 abstracts and select the articles that would be included in the study based on the selection criteria. Afterwards, the selected articles by the two authors were compared and there was complete concurrence on the category “yes, this one will be included”. For some of the articles that were marked as “maybe”, first and second author had a brief discussion to reach a consensus. This helped to reach higher reliability for the inclusion of the articles. Further in the process, the second author consulted the first author whenever there was a doubt whether to include or exclude the article. In addition, regular meetings with the third author also contributed to the overall process of the selection.

### Data analysis

The resulting papers were characterized by the research aim and the type of research, which is reflected in the Table 1. The papers were further categorized according to the focus of the research question and data. Each paper’s empirical findings were categorized by looking at data and making first notes inductively. Following this, we looked at our notes on topics that emerged from analysed articles and compared them to earlier literature. In this way, concepts from prior literature helped us to make the sense of data from different articles and categorize them. A good example for that is the concept of social support, which we used to classify types of use. After analysing the articles in this way, we formulated propositions in the discussion section.

**Table 1:** Overview of included studies in the literature review

Year	Author(s) - Article no.	Journal	Main objective of study	Type of research	Data collection	Participants (sample)
2005	[13]	Journal of Sociology	To explore the experiences of, and attitudes towards, online support groups	Qualitative	Interviews	33 Australian men with prostate cancer and 18 specialists
2008	[22]	Journal of Medical Internet Research	To explore whether lurkers in online patient support groups profit to the same extent as posters do	Quantitative	Online survey	528 members of Dutch online support groups for patients with breast cancer, fibromyalgia, and arthritis
2008	[28]	Journal of Medical Internet Research	To identify and analyse how users of the platform PatientsLikeMe reference personal health information within patient-to-patient dialogues	Qualitative	Analysis of comments	123 comments posted within the ALS community
2010	[15]	New Review of Hypermedia & Multimedia	To understand why and how people use health-related sites	Quantitative	Online survey	33 Patients with a medical condition (patients)
2010	[27]	Pedriatic Transplantation	To investigate the feasibility and safety of an online virtual community as a potential psychosocial intervention for post-transplant adolescents	Qualitative and Quantitative	Data analysis of the Zora system logs and interviews	22 patients with solid organ transplants aged between 11-15 years
2010	[35]	Journal of Psychosomatic Obstetrics & Gynecology	To focus on investigating the perceived disadvantages of online infertility support communities from the perspective of those who access and participate in them	Qualitative and Quantitative	Online survey	295 participants coping with fertility problems
2010	[36].	Journal of Medical Internet Research	To describe the potential benefits of PatientsLikeMe in terms of treatment decisions, symptom management, clinical management, and outcomes	Quantitative	Online survey	1323 members from six PatientsLikeMe communities (ALS, MS, Parkinson’s Disease, HIV, fibromyalgia, and mood disorders)
2011	[23]	Patient Education and Counseling	To investigate the potential of online support groups to foster empowerment and how membership might affect the patient/health professional relationship	Quantitative	Online survey	246 individuals from 33 chronic conditions online support groups
2011	[26]	Journal of Medical Internet Research	To explore the differences in peer support received by lurkers and posters in online breast cancer communities	Quantitative	Online survey	253 members of four Japanese online breast cancer communities
2012	[16]	Journal of Medical Internet research	To explore the motivations and challenges faced by patients who share videos about their health and experiences on YouTube	Qualitative	Analysis of videos	Videos uploaded by 4 patients with a chronic condition
2012	[30]	Health Communication	To examine the indirect effect of Computer Mediated Social Support on doctor–patient communication through utilizing the sense of empowerment	Quantitative	Online survey	464 Korean patients with diabetes
2012	[38]	Information Research	To examine the use of an online health forum by married Korean women living in the USA who sought help for health and medical issues	Qualitative	Content analysis of posts	1000 messages posted to a health forum MissyUSA
2013	[14]	International Journal of Medical Informatics	To investigate whether communication in online patient support groups is a source of individual as well as collective empowerment or to be understood within the tradition of compliance	Qualitative	Analysis of posts	4301 posts from two online communities, one for patients with COPD and one for women with pregnancy problems
2013	[24]	Journal of Health Psychology	To explore how cancer patients’ writing and reading on the Internet play a role in their conditions experience	Qualitative	Focus-group interviews	34 Cancer patients
2013	[25]	JRSM short reports	To explore how participation in an online support community may impact upon the experience of inflammatory bowel disease	Qualitative and Quantitative	Online survey	249 patients living with either Crohn’s Disease (65.9 %) or Ulcerative Colitis (26.1 %) or awaiting formal diagnosis (8 %)
2013	[34]	Nordic Journal of Psychiatry	To evaluate if and how online self-help forums are used by patients with bipolar disorders, their relatives and treating professionals	Qualitative and Quantitative	Content analysis of posts	2400 postings of 218 users (Patients with Bipolar Disorder (94 %), Relatives (4 %), or Professionals (2 %))
2014	[1]	Patient Education & Counseling	To explore how individuals use online health community content in clinical discussions and how healthcare providers react to it	Qualitative	Focus groups	89 members of an online health community
2014	[17]	Obstetrics & Gynecology	To determine whether social media, specifically Facebook, is an effective tool for improving contraceptive knowledge	Quantitative	Survey	143 Patients who had scheduled a routine visit to a gynaecologist
2014	[21]	Indian Journal of Psychological Medicine	To explore the potentials of social networking sites as an adjunctive treatment modality for initiating treatment contact as well as for managing psychological problems	Qualitative and Quantitative	Interviews and an online survey	28 patients with any of the depressive or anxiety spectrum disorder
2014	[37]	Reproductive Health	To use the online platform of blogs to explore whether the framing effect of information content, situated learning of information content, and health knowledge involvement would affect health communication between doctors and patients and further explore whether this would increase patient willingness to seek treatment	Quantitative	Online survey	278 participants who were seeking medical treatment in a clinic or hospital in Taiwan
2014	[39]	Journal of the American Medical Informatics Association	To describe adults who use Twitter during a weight loss attempt and to compare the positive and negative social influences they experience from their offline friends, online friends, and family members	Qualitative and Quantitative	Survey	100 participants trying to lose weight
2016	[40]	Counselling Psychology Quarterly	To test for differences between offline and online psychological disclosure in case of young adults	Quantitative	Survey	128 young adults attending individual psychotherapy.

## Results

### Search results

The searches were carried out in the period ending on March 17th, 2015. The application of the search strategy to the two search engines resulted initially in a total of 1,743 articles. Within the 1,743 articles many duplicates were found as well within the search engines as between the search engines. By removing duplicates the first found article was kept. In this way, we identified and removed 468 duplicates leaving us with 1,275 articles.

The remaining 1,275 articles were screened on title and abstract with regards to the selection criteria. Whenever we had doubts if an article is relevant or when title and abstract were not clear, we inspected the paper in more details by accessing full article. An article was removed when, for example, it became clear that the user of social media was not a patient but another user, like the hospital, a regular “healthy” person or healthcare professional. Additionally, several articles referred to internet use by patients for health related reasons and their effects, but did not specify the effects of social media. Therefore, such articles were removed. Moreover, articles that were written in a language other than English as well as articles that did not comprise primary data or did not elaborate on an effect of patients using social media. This left us with 22 articles that met our criteria. In addition, as a result of the referees’ suggestion to include term “client”, we identified one additional article, making the entire list of 23 articles for the quality assessment.

Quality of the articles was assessed by using the Standard Quality Assessment Criteria for Evaluating Primary Research Papers by [12] as presented in the Appendix C (See Additional file 3). This assessment tool distinguishes between qualitative and quantitative research and provides different quality assessment criteria for each type of research. The criteria are rated on their presence in the respective article and are either completely addressed in the article (resulting in 2 points), partly addressed (resulting in 1 point), or not addressed (resulting in 0 points). In case an article scored below the threshold of a 50 % score of the total amount of points possible, the article is assumed to be of low quality and removed from this paper. This cut-off point for inclusion is relatively liberal according to the authors of the assessment tool [12]. One article had a quality score below the 50 % cut-point and was excluded, which left us with the total of 22 articles for analysis.

**Fig. 1:**
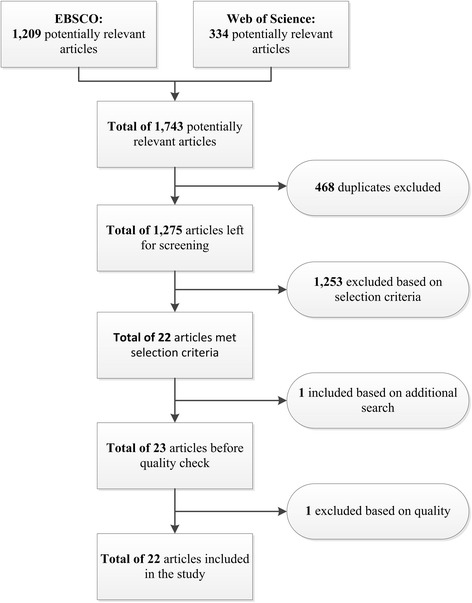
Flowchart of study selection process

The article selection process is shown in Fig. 1.

### Overview of the articles

The Table 1 provides an overview of 22 articles included in the study. All studies except for three were published in or after 2010. Moreover, 19 articles were published in journals that are related to the medical field, whereas only three articles are published in journal that do not have a specific connection to medicine: Journal of Sociology, New Review of Hypermedia & Multimedia, and Information Research. Only two out of the 22 articles use a theory or a model to build their research on, namely the concept of masculinity [13] and the actant model [14]. The group of articles consists of nine quantitative, seven qualitative and six mixed methods studies.

The analysis of articles with regard to the type of social media and conditions is presented in the Appendix D (See Additional file 4), which shows that the 12 articles studied online support communities and most focused on chronic conditions. Other types of social media platforms and conditions were spread among the remaining articles.

### Analysis of results

This section presents findings from 22 articles we included in our study. First of all, an overview of the extracted findings is presented regarding the types of social media use by patients. Following this, we present the effects of social media use on patients. Subsequently, an overview of the extracted findings regarding effects of social media use by patients on the relationship between patients and healthcare professionals are presented, discussed, and categorized.

#### Types of social media use by patients for health related reasons

Our analysis starts with the type of use and motivation for their use of social media. When analysing all articles it becomes clear that patients do not use social media to circumvent healthcare professionals, but rather use it as a complement to healthcare professional services to fulfil the patients’ needs that cannot be met by the healthcare professional. The relationship between patients and healthcare professionals is viewed by the patients as a more clinical one, where healthcare professionals provide expert knowledge about the condition and recommend treatment based on their medical knowledge, but not on their first-hand experience [15].

Additionally, doctors often have difficulty expressing empathy and that they filter information for the patient, where the patient would rather be informed about all options. Patients also believe that doctors might not be aware of the latest breakthroughs [15]. Moreover, one of the the main reasons for patients to join online health communities is their dissatisfaction with their healthcare professional’s inability to meet the patients’ emotional and informational needs [1]. Another reason for patients to use social media was to bridge the gap between traditional health information about their condition and everyday life [16]. In particular, Facebook is seen as an important addition to traditional in-office counselling in improving patient knowledge [17].

Therefore, the types of social media use by patients as identified in this paper refer to the way in which patients use social media intended to meet an unfulfilled need. These are identified in the articles are categorized as shown in Table 2 and explained below. Categories represent social support, consisting of emotional, esteem, informational, and network support [18], and other types of use, which are emotional expression and social comparison.

**Table 2:** Types of use of social media by patients for health related purposes by article

Type of use		Article no.
Social support	Emotional support	[1, 13, 16, 21–23, 25, 26, 30, 34–36, 40]
	Esteem support	[14, 16, 23–25, 30, 39]
	Information support	All articles
	Network support	[1, 14–16, 21, 24–28, 34, 36, 39]
Other types of use	Emotional expression	[13–15, 21, 24–26, 38]
	Social comparison	[23, 25, 35, 39]

##### Social support

The most common type of social media use by patients for health related reasons that we found is social support. Social support is defined as “the process of interaction in relationships which is intended to improve coping, esteem, belonging, and competence through actual or perceived exchanges of psychosocial resources” [19]. Social support is represented through five different categories and four of these categories were found to be common types of social media use by patients for health related purposes [18]. These four types, namely emotional support, esteem support, information support, and network support are explained below.

*Emotional support*. Emotional support is defined as “communication that meets an individual’s emotional or affective needs” [20]. It refers to support gained through expressions of care and concern, which serve to improve an individual’s mood. Emotional support helps patients to meet their emotional or affective needs. The use of social media by patients for emotional support was identified in 13 articles. Examples of emotional support are “sharing of emotional difficulties” [21], “encountering support that feels like a warm blanket wrapped around you” [22], and “share emotions with other people who are coping with similar problems” [23].

*Esteem support*. Esteem support refers to “communication that bolsters an individual’s self-esteem or beliefs in their ability to handle a problem or perform a needed task” [20]. The aim of this type of support is to encourage individuals to take the actions needed to successfully live with their condition. The use of social media by patients for esteem support was identified in seven articles. Examples of esteem support include “getting support from other patient’s encouragement” [24], “share experiences about a new treatment to find encouragement before starting it” [25], and “rituals of confirming each other’s endeavours to follow health instructions” [14].

*Information support*. Information support is “communication that provides useful or needed information” [20]. In particular, newly diagnosed patients are in a need for a lot of information about their condition and treatment options, which can be provided by patients who have already dealt with the condition for a longer period [20]. The use of social media by patients for information support was identified in all articles. Examples of information support are “receiving advice about treatments” [26], “help fellow sufferers by sharing experiences and relevant information about the disease” [24], and “ask questions about the condition” [25].

*Network support*. Network support is defined as “communication that affirms an individual’s belonging to a network or reminds him/her of support available from the network” [20]. Hence, network support is support that reminds people that no matter what situation they are facing, they are not alone. The use of social media by patients for network support was identified in 13 articles. Examples of network support include “meeting other patients who had gone through similar experiences” [27], “a means to connect with others in similar situations” [15], and “fostering relationships based on shared attributes” [28].

##### Other types of use

In addition to the social support, we also identified two other types of use, which could not be directly placed under one of the subcategories of social support. These are emotional expression and social comparison.

*Emotional expression*. Emotional expression refers to the unique opportunity provided by social media for patients (and other users) to express their emotions freely without having to be concerned about the immediate feelings or reactions of those who stand close to them. As noted in one of the articles, “online communities provide the potential to allow patients to open up and reduce the inhibitions felt in sharing experiences in face to face situations”, e.g. hurting other people’s feelings [13]. Therefore, patients can use social media as a place to express their emotions freely, like, releasing negative emotions [24]. In contrast to emotional support, which is defined as patients interacting in and receiving communication to meet their affective needs, emotional expression refers to patients expressing their emotions regardless of whether someone will respond. The use of social media by patients for emotional expression was identified in 8 articles. Examples include “a place to vent about the illness” [25] and “an outlet for expressing your emotions freely” [15].

*Social comparison*. Patients use social media to compare themselves with other patients to see how “bad” their condition is or to find out how the treatments work. This social comparison can seem to overlap with social support, for instance, when patients compare themselves to peers to recognize that they are not the only person in this situation (network support) or when patients compare themselves to peers to find out how other people suffer from or cope with the condition (esteem support, emotional support, or information support). However, social comparison was categorized separately as within the articles the authors presented it as a different type of use without specifying the details. The use of social media by patients for social comparison was identified in four articles. Examples include “upward social comparison” [25] and “comparison with other members [23].

#### Effects of the different types of social media use by patients on patients

In this section the effects of the use of social media by patients for health related reasons are analysed and presented. The most common effect of patients using social media for health related reasons is patient empowerment, which is represented through three categories: enhanced subjective well-being, enhanced psychological well-being, and improved self-management and control. We also identified four other types of effects, which are less common in our literature review. These are: diminished subjective well-being, loss of privacy, addiction to social media, and being targeted for promotion. Identified categories are presented in Table 3 and explained below.

**Table 3:** Effects of social media use by patients for health related reasons by article

Effect		Article no.
Patient empowerment	Enhanced subjective well-being	[13, 15, 21–27, 30, 36, 39]
	Enhanced psychological well-being	[13–16, 21–25, 27, 28, 34, 39, 40]
	Improved self-management and control	[14–17, 22–26, 28, 30, 34, 36–38]
Other types of effects	Diminished subjective well-being	[13, 16, 25, 26, 35, 36]
	Loss of privacy	[16]
	Being targeted for promotion	[16]
	Addiction to social media	[35]

##### Patient empowerment

In current literature, the concept of empowerment is defined as “an individual trait, characterized by an emphasis on increased individual control over the aspects of one’s life” [29]. We argue that the patient empowerment refers to “the discovery and development of one’s inherent capacity to be responsible for one’s own life. Hence, patients are empowered when they are in possession of the knowledge, skills, and self-awareness necessary to identify and attain their own goals” [14]. Information support, esteem support, and emotional support were significant predictors of a patient’s sense of empowerment [30]. Informational support was the strongest predictor of increased sense of empowerment followed by esteem support and emotional support. The three subcategories of empowerment, namely enhanced subjective well-being, enhanced psychological well-being, and improved self-management and control, are discussed below.

*Enhanced subjective well-being*. Subjective well-being refers to “what people think and how they feel about their lives in positive ways” [31]. In this paper, enhanced subjective well-being mainly refers to the pleasant emotions patients experience due to their social media use for health related reasons. “People experience enhanced subjective well-being when they feel many pleasant and few unpleasant emotions” [31]. Consequently, enhanced subjective well-being refers to an increase in the experience of pleasant emotions, which in turn heightens people’s feeling of empowerment. The effect enhanced subjective well-being was identified in 12 articles. Examples from the articles concerning enhanced subjective well-being are “increased optimism” [22], “increased acceptance of the illness” [23], “decrease anxiety” [26] and “increased sense of normalcy” [27].

*Enhanced psychological well-being*. Psychological well-being is defined in the literature as “focusing on eudemonic well-being, which is the fulfilment of human potential and a meaningful life” [32]. One of the components affecting psychological well-being is the experience of positive relations with others. It is argued that a central component of mental health is to be in warm, trusting, interpersonal relations [33]. Moreover, “self-actualizers are described as having strong feelings of empathy and affection for all human beings and as being capable of greater love, deeper friendship, and more complete identification with others” [33]. Therefore, enhanced psychological well-being refers to an increase in the patient’s experience of positive relations with others through the use social media. The effect enhanced psychological well-being was identified in 14 articles. Examples from the articles include “feeling of being connected to other people” [34], “increased social network online as well as offline” [27], and “promotion of deep relationships” [15].

*Improved self-management and control*. Improved self-management and sense of control refers to the improvement in the capability of patients to better handle their condition. As patients feel better informed, their ability to make decisions on their own improves, which fosters self-management and perceived control over the condition. Ability to deal with the day-to-day life with the condition also increases, for example due to learning about coping strategies, which also fosters improved self-management and perceived control. The effect of improved self-management and sense of control was identified in 14 articles. Examples from the articles include “increase patient’s self-management” [34], “improvement in the ability to manage the disease” [16], and “fostering insight and universality” [26].

##### Other types of effects

In addition to the patient empowerment, several other types of effects of social media use by patients on patients were identified. These are diminished subjective well-being, loss of privacy, being targeted for promotion, and addiction to social media.

*Diminished subjective well-being*. Diminished subjective well-being is opposite of enhanced subjective well-being and indicates an increase in the experience of negative emotions due to the use of social media, such as an increase in feelings of worry and anxiety. It was identified in six articles. Diminished subjective well-being was the most common found effect of patients using social media for health related reasons. Examples include “demoralization” [25], “hurt feelings due to negative feedback” [16], and “increased feelings of anxiety” [35].

*Loss of privacy*. Loss of privacy was mentioned in only one article [16]. It refers to the finding that the patients lose their privacy when they post personal videos on YouTube.

*Being targeted for promotion*. Being targeted for promotion was also mentioned in only one article by [16]. It refers to the finding that patients who post videos on YouTube can be targets product promotions.

*Addiction to social media*. Addiction was an effect identified in one article by [35]. It refers to the finding that sometimes patients experience their social media use for health related reasons to be addictive. As such, it often took the time that they usually spent doing other tasks.

#### Effects of social media use by patients on the relationship between patients and healthcare professionals

The use of social media by patients for health related reasons does not only affect the patients themselves or other patients, but also the relationship between patients and healthcare professionals. In total, nine articles discussed the effects of social media use by patients on the relationship between patients and healthcare professionals, although six out of these nine articles only touch very briefly upon this subject. The effects of social media use by patients for health related reasons on the relationship between patients and healthcare professionals that have been extracted from the articles are presented in Table 4 and discussed below.

**Table 4:** Effects of social media use by patients on the healthcare professional – patient relationship

Effect		Article no.
Healthcare professional-patient relationship	More equal communication	[22, 23, 30, 36, 37]
	Switching of doctors	[1, 36]
	Harmonious relationship	[14, 24]
	Suboptimal interaction	[1, 13]

The findings presented in Table 4 are divided into categories representing the effects on the relationship between patients and healthcare professionals. These categories are more equal communication between the patient and healthcare professional, increased switching of doctors, harmonious relationships, and suboptimal interaction between the patient and healthcare professional. The categories are discussed below.

##### More equal communication between the patient and healthcare professional

Social media use by patients for health related reasons can lead to more equal communication between the patient and healthcare professional. This effect refers to patients feeling more confident in their relationship with the healthcare professional. In total, five articles referred to this effect. With the information from the social media platforms, patients can increase their knowledge about treatment options. Consequently, they are better able to communicate with the healthcare professional as they can better understand their condition [36]. Hence, patients may feel more confident in their relationship with their physician [22, 23]. Patients feel that they are better prepared for consultations as they are more informed about their condition and know better what questions to ask [23]. Social support received through the use of social media eventually increases the likeliness to form an intention to actively communicate with the doctor during a medical consultation [30]. Moreover, the use of social media provides the opportunity to learn and increase health communication, which may lead to an increase in the patients’ willingness to seek medical attention [37]. Hence, these findings suggest that the use of social media for health related purposes can increase a patient’s confidence and active communication in their relationship with healthcare.

##### Increased switching of doctors

Social media use by patients for health related reasons can lead to shorter relationships between healthcare professionals and patients. Patients may change doctor due to online discussions about physicians or due to negative reactions from doctors about the patients’ treatments supervised by their regular physicians. Two articles found that patients changed physician because of those patients’ use of social media. For example, negative reactions from physicians to the mentions of social media use by patients made the patients to look for second opinion and even change their doctor [1]. On the other hand, some patients changed their doctor as a result of online discussion with other patients [36].

##### Harmonious relationships

Harmonious relationships between healthcare professionals and patients can be established as social media provide a place for patients to release negative emotions. However, the effect of harmonious relationships also comprises the fact that social media might empower individuals to follow doctor’s recommendations, which reduces discussions during clinical interaction. The effect of harmonious relationships was identified in two articles. Social media provide a place for patients to express their emotions and maintain harmony in the relationship between healthcare professional and patient in offline consultations, which focuses on non-emotional aspects of the disease [24]. On the other hand, social media were empowering individual users to comply with doctors’ recommendations as a group, which affects the healthcare professional patient relationship by potentially reducing discussions during clinical interactions as patients stick to the recommended treatment [14]. However, it can also be viewed as a missed opportunity, as patients do not empower each other to find alternative treatments [14].

##### Suboptimal interaction between the patient and healthcare professional

As patients use social media for health related reasons, this can affect the patient and healthcare professional relationship by leading to suboptimal interaction between the patient and healthcare professional. When patients bring social media content to the consultation, this can lead to increased processes of sorting information, transforming the potential risk to the healthcare professional, and challenging the healthcare professional’s expertise [13]. Additionally, if the healthcare professional reacts negatively to what patient learned from social media, this might decrease the patient’s subjective well-being [1]. The effect of suboptimal interaction between the patient and healthcare professional was identified in two articles. Discussion of the information from social media during the consultation was experienced as a threat by the physician [13]. Furthermore, healthcare professionals reacted negatively to online health community content raised during clinical interactions, which made patients feel disempowered, but it did not change their online behaviour [1].

#### Relationship between effects on patients and effects on the patient healthcare professional relationship

In the section about the effect of “more equal communication between the patient and healthcare professional”, we already mentioned that increased communication during a consultation on behalf of the patient can be caused by patient empowerment. Patient empowerment refers to “the inherent capacity to be responsible for one’s own life” [14]. In regards to the relationship between patients and healthcare professionals, the patients took more responsibility for their own condition. Five articles find that the patient empowerment indeed affects the patients’ confidence, ability and willingness to actively participate in clinical interactions. Patients increased their sense of empowerment through their intention to actively communicate with the doctor [30]. Additionally, the patient empowerment was associated with an increased confidence in dealing with the physician [23]. Moreover, the convenience of social media use by patients is that it reduces the information gap between healthcare professionals and patients and patients have a better understanding of the healthcare professional during consultations [37]. Social media can empower patients by giving them access to information and opportunities for discussions, which increases the patient’s involvement in clinical interactions [15]. Finally, the patient empowerment increases the ability of patients to communicate with the healthcare professionals [22]. Hence, we argue that the patient empowerment contributes to more equal communication between the patient and the healthcare professional.

## Discussion

This review provides an insight into the current body of knowledge on the effects of social media use by patients for health related reasons and the effects on patients and on their relationship with healthcare professionals. All of the studies were published in the past 10 years, with only three articles published before 2010. This can be explained by a recent increase in the use of social media by patients for health related reasons.

We categorized articles into different types of use and effects. We identified that the most common type of use was social support, namely emotional support, esteem support, information support, and network support. The types of social media use were most often found to affect patients by empowering them through enhanced subjective well-being, enhanced psychological well-being, and improved self-management and control. However, the types of social media use by patients were also found to affect patients through addiction to social media, diminished subjective well-being, being targeted for promotion, and loss of privacy. Moreover, the identified types of social media use by patients for health related reasons was also found to affect the relationship between patients and healthcare professionals as it can result in more equal communication between the patient and healthcare professional, shorter relationships, harmonious relationships, and suboptimal interaction between the patient and healthcare professional. Based on these findings, we made three propositions.

### Relationship between use and effect: Network support and enhanced psychological well-being

When patients are diagnosed with a certain condition that nobody in their close (offline) network has experienced before, patients can feel very lonely [27]. As a diabetic patient states “I literally felt like the only diabetic on the planet” [16]. However, social media provide an opportunity to easily connect with others and reduce this feeling of loneliness. Consequently, patients using social media for network support enhanced their psychological well-being. For example, social media provide means to connect with others in similar situations and this can break a patient’s loneliness [15]. This is in line with earlier studies that have shown how the existence of network support contributes to a better well-being of the patients [41, 42]. Interestingly, [41] suggest that the network support may not only benefit the patients themselves, but also their families who care for them. Yet, the relationship between the network support and psychological well-being may depend on the level of self-esteem. For example, college students with low self-esteem profited more from online social networking sites for bridging social capital and starting relationships than college students with high self-esteem [43]. In line with that, social networking sites provides the unique opportunity for patients to be able to talk about the sensitive aspects of the condition, as online communities provide the potential to reduce inhibitions felt in sharing experiences face to face [13]. Such an inhibition could reflect low self-esteem in terms of a reluctance to talk about the condition in face to face conversations.

*Proposition 1: Social media use by patients for network support leads to enhanced psychological well-being. This effect is stronger for people with low self-esteem than for the people with high self-esteem.*

### Relationship between content and effect: Reading other people’s stories, improved self-management and control and enhanced subjective well-being

Not all patients that make use of social media use it actively. Sometimes patients only use social media to read about other people’s stories, without actively contributing themselves. These people are called lurkers. The lurking behaviour may be related to the level of privacy concerns and computer anxiety [44]. In particular, anxiety leads to increase in lurking. Two articles in our sample were focused on the effects of patients using social media merely by reading other people’s stories. From the two articles, it becomes clear that the effects experienced by reading other people’s stories are being better informed [22, 26]. Additionally, by reading other people’s stories anxiety was found to significantly decrease [26]. Consequently, these findings suggest that reading other people’s stories on social media can lead to enhanced subjective well-being and improved self-management and control. However, [22] and [26] do not elaborate on the content of the stories read. Contrasting findings were found in other articles regarding how content affects the effects of reading other people’s stories. For example, cancer patients who read other people’s stories enhanced their subjective well-being [24]. Reading about success stories was found to enhance confidence to fight the condition, whereas reading about bad experiences prepared the patient mentally for difficult times ahead. On the other hand, the patients suffering from an inflammatory bowel disease who read other people’s stories about a bad experience suffered from diminished subjective well-being [25]. This is in line with earlier findings showing that the lack of sharing and feedback on this sharing may threaten the need for belonging [45]. Finally, patients suffering from infertility experienced diminished subjective well-being as the result of reading other people’s stories [35]. Reading stories about successful pregnancies led to increased feelings of jealousy, pain and a sense of alienation, whereas reading about bad experiences led to increased feelings of worry, anxiety and decreased optimism. Thus, this may lead to diminished subjective well-being. On the other hand, one study in our sample shows that this actually may enhance subjective-well-being [24]. In particular, this paper focused on blogs whereas other studies focused on online support groups [24]. Among other uses, blogs can be used as personals diaries to express thoughts, feelings, and stories [9]. Level of distress actually decreases when people blog about their emotional difficulties [46].

*Proposition 2: Reading other people’s stories about a negative experience leads to diminished subjective well-being. This effect is weaker for patients who blog about their experiences than for those who do not.*

### Relationship between patients and healthcare professionals: shift in power balance and increased quality of decision making

The effects of social media use by patients for health related reasons show that social media use by patients can lead to patient empowerment. Patient empowerment is an established concept in the medical research and has been promoted to foster patient autonomy [47]. As a result of the patient empowerment, patients may increasingly interact with their healthcare professional and get more involved in the decision making process [15]. In this case, social media can be seen as a “new” technology adopted by patients, which may shift the power balance between the healthcare professional and the patient. The use of new technologies in healthcare has been suggested as a way to empower end-consumers by enabling speed and convenience in accessing health related information [48]. In this line, the patients are able to actively participate in the interactions with healthcare professionals. On the other hand, the healthcare professionals may experience a decrease in power in the decision making process. According to the political variant of the interaction theory [49], “a product of the interaction of system features with the intra-organizational distribution of power, defined either objectively, in terms of horizontal or vertical power dimensions, or subjectively, in terms of symbolism can be resistance to the system”. Hence, redistribution of power between patients and healthcare professionals may cause the resistance from healthcare professionals. Yet, the role of health professionals has to change because embracing patient empowerment in healthcare means making a change, which sometimes seem difficult due to traditional approach, which is embedded in their current training [50].

However, increased patient involvement in the clinical interaction could potentially increase the risk placed on the healthcare professionals [13]. Healthcare professional may not be in complete control of the information used during decision making as the patient also has a voice, but the healthcare professional bears full responsibility for the decision taken. When patients bring in the information from social media to the consultation, this could lead to unnecessary processes of sorting relevant information from irrelevant information and can be experienced as challenging the healthcare professional’s expertise [1, 13]. Hence, based on these findings it is possible for healthcare professionals to resist this shift in the balance of power. However, increased equalization of the healthcare professional and patient communication can be a positive and desired effect. In particular, healthcare professionals may become more patient-centred, thus complementing the patient empowerment [51]. As a consequence of patient empowerment, we propose that the quality of clinical decision making may be enhanced.

According to the concept of bounded rationality [52], not all information can be gained on all available treatment options by healthcare professionals, as the human mind has a limited capacity to process the available information and often time is limited as well. Hence, healthcare professionals are unable to know all the information regarding treatment options and the newest developments, which affects their decision making. Thus, patients can extend this information base of the healthcare professional by specializing themselves in their own condition. This could provide an opportunity to increase the quality of the treatment decisions.

*Proposition 3: As a result of patient empowerment due to patients using social media for health related reasons, the power balance between healthcare professionals and patients becomes more equalized, leading to increased quality of clinical decisions making.*

Notwithstanding the interesting results described above, this research has some limitations which, along with the three propositions, suggest opportunities for further research. It is possible that we missed some articles that could have used different terminology. Consequently, the results of this paper might not be generalizable for all social media platforms. For practical reasons, we excluded non-English papers. Finally, a limitation of every literature review is that the authors of the included articles will have had different objectives and used different methods and means of interpretation in reaching their conclusions. In this paper, we highlighted the most important findings on our topic of study and we categorized the key effects of social media use on patients and on their relationships with healthcare professionals.

## Conclusions

The use of social media by patients for health related reasons is growing. This systematic literature review reflects on beneficial and potentially harmful effects of social media use by patients for health related. The findings show that patients use social media mainly for social support, which is represented through information support, emotional support, esteem support, and network support. Other identified types of social media use by patients have found to be emotional expression and social comparison. These types of social media use by patients were found to most commonly lead to patient empowerment. Other effects of social media use by patients we identified were diminished subjective well-being, addiction to social media, being targeted for promotion, and loss of privacy. The types of social media use by patients were also found to affect the healthcare professional and patient relationship by stimulating more equal communication between the patient and healthcare professional, shorter relationships, harmonious relationships, suboptimal interaction between the patient and healthcare professional. Whereas some of the articles discussed the effects of patients’ use of social media on relationship between patients and healthcare professionals briefly, we encourage future research to tackle this issue. We developed three propositions, which may also stimulate further research in this respect.

## Additional files

Additional file 1: Appendix A-Search string. (DOCX 14 kb) Additional file 2: Appendix B-List of databases. (DOCX 14 kb) Additional file 3: Appendix C-Quality assessment [53]. (DOCX 21 kb) Additional file 4: Appendix D-Summary of articles per social media category. (DOCX 14 kb)
