# Supplementary material for: Social media use in healthcare: A systematic review of effects on patients and on their relationship with healthcare professionals
Source: BMC Health Serv Res. 2016 Aug 26;16(1):442. doi: 10.1186/s12913-016-1691-0 (PMC5000484; doi:10.1186/s12913-016-1691-0)
Supplement: Additional file 1: — Appendix A-Search string. (DOCX 14 kb) [file 12913_2016_1691_MOESM1_ESM.docx]

**Appendix A – Search string**

| Search Line | Terms used* | Fields | Search Engine |
| --- | --- | --- | --- |
| 1 | “social media” OR blog* OR “content communit*” OR “social networking site*” OR “online social network*” OR “virtual world*” OR “online communit*” OR “online forum*” OR Facebook OR Twitter OR Wikipedia OR IMVU OR “second life” OR YouTube | Not selected | EBSCO |
| 2 | “Patient*” | Not selected | EBSCO |
| 3 | “health* provider*” or “health* professional*” or “physician*” or “doctor*” or “hospital*” | Not selected | EBSCO |

| Search Line | Terms used* | Fields | Search Engine |
| --- | --- | --- | --- |
| 1 | “social media” OR blog* OR “content communit*” OR “social networking site*” OR “online social network*” OR “virtual world*” OR “online communit*” OR “online forum*” OR Facebook OR Twitter OR Wikipedia OR IMVU OR “second life” OR YouTube | Topic | Web of Science |
| 2 | “Patient*” | Topic | Web of Science |
| 3 | “health* provider*” or “health* professional*” or “physician*” or “doctor*” or “hospital*” | Topic | Web of Science |

***As suggested by the referees of this paper*,*we also used the terms*“*client**”* (in addition to patient). This yielded one additional article.
