# Supplementary material for: Social media use in healthcare: A systematic review of effects on patients and on their relationship with healthcare professionals
Source: BMC Health Serv Res. 2016 Aug 26;16(1):442. doi: 10.1186/s12913-016-1691-0 (PMC5000484; doi:10.1186/s12913-016-1691-0)
Supplement: Additional file 2: — Appendix B-List of databases. (DOCX 14 kb) [file 12913_2016_1691_MOESM2_ESM.docx]

**Appendix B – List of databases**

This appendix provides an overview of all the databases that are covered by the search engines. The databases that are in italic were excluded from the search as these databases did not touch upon the fields of social media or healthcare

**Web of Science**1) Web of Science Core Collection
2) Biological Abstracts
3) Data Citation Index
4) KCI-Korean Journal Database
5) MEDLINE
6) SciELO Citation Index

**EBSCO**
1) Academic Search Premier
2) AMED - The Allied and Complementary Medicine Database
3) *America: History & Life*
4) *American Bibliography of Slavic and East European Studies*
5) *Arctic & Antarctic Regions*
6) *Art Full Text (H.W. Wilson)*
7) *Art Index Retrospective (H.W. Wilson)*
8) *ATLA Religion Database with ATLASerials*
9) Business Source Premier
10) CINAHL
11) Communication & Mass Media Complete
12) *eBook Academic Collection (EBSCOhost)*
13) *eBook Collection (EBSCOhost)*
14) *EconLit*
15) ERIC
16) *GreenFILE*
17) *Historical Abstracts*
18) *L'Année philologique*
19) Library, Information Science & Technology Abstracts
20) MEDLINE
21) *Military & Government Collection*
22) *MLA Directory of Periodicals*
23) *MLA International Bibliography*
24) *New Testament Abstracts*
25) *Old Testament Abstracts*
26) *Philosopher's Index*
27) PsycARTICLES
28) *PsycBOOKS*29) *PsycCRITIQUES*
30) PsycINFO
31) *Regional Business News*
32) *RILM Abstracts of Music Literature*
33) SocINDEX
