# Supplementary material for: Social media use in healthcare: A systematic review of effects on patients and on their relationship with healthcare professionals
Source: BMC Health Serv Res. 2016 Aug 26;16(1):442. doi: 10.1186/s12913-016-1691-0 (PMC5000484; doi:10.1186/s12913-016-1691-0)
Supplement: Additional file 3: — Appendix C-Quality assessment [53]. (DOCX 21 kb) [file 12913_2016_1691_MOESM3_ESM.docx]

**Appendix C – Quality assessment**

**Table 1: Quality assessment qualitative studies**

| **Criteria quantitative studies** | **[15]** | **[17]** | **[22]** | **[23]** | **[26]** | **[30]** | **[36]** | **[37]** | **[40]** |
| --- | --- | --- | --- | --- | --- | --- | --- | --- | --- |
| Question/objective sufficiently described? | 2 | 2 | 2 | 2 | 2 | 2 | 2 | 2 | 2 |
| Study design evident and appropriate? | 2 | 2 | 2 | 2 | 2 | 2 | 2 | 2 | 2 |
| Method of subject/comparison group selection or source of information/input variables described and appropriate? | 2 | 2 | 2 | 1 | 2 | 1 | 2 | 2 | 2 |
| Subject (and comparison group, if applicable) characteristics sufficiently described? | 1 | 2 | 2 | 1 | 2 | 2 | 1 | 1 | 2 |
| If interventional and random allocation was possible, was it described? | N/A | N/A | N/A | N/A | N/A | N/A | N/A | N/A | N/A |
| If interventional and blinding of investigators was possible, was it reported? | N/A | N/A | N/A | N/A | N/A | N/A | N/A | N/A | N/A |
| If interventional and blinding of subjects was possible, was it reported? | N/A | N/A | N/A | N/A | N/A | N/A | N/A | N/A | N/A |
| Outcome and (if applicable) exposure measure(s) well defined and robust to measurement/misclassification bias? Means of assessment reported? | 1 | 2 | 2 | 1 | 2 | 2 | 2 | 1 | 1 |
| Sample size appropriate? | 1 | 2 | 2 | 2 | 2 | 2 | 2 | 2 | 2 |
| Analytic methods described/justified and appropriate? | 1 | 2 | 1 | 1 | 2 | 2 | 1 | 1 | 2 |
| Some estimate of variance is reported for the main results? | N/A | 0 | 2 | 2 | 0 | 2 | 0 | 2 | 2 |
| Controlled for confounding? | N/A | 1 | 1 | 2 | 2 | 2 | 2 | 0 | 0 |
| Results reported in sufficient detail? | 1 | 2 | 2 | 2 | 2 | 2 | 2 | 2 | 2 |
| Conclusions supported by the results? | 2 | 2 | 2 | 2 | 2 | 2 | 2 | 2 | 2 |
| **Total score/possible maximum score** | **13/18** | **19/22** | **20/22** | **18/22** | **20/22** | **21/22** | **16/22** | **17/22** | **19/22** |

| **Criteria qualitative studies** | **[1]** | **[13]** | **[14]** | **[16]** | **[24]** | **[28]** | **[38]** | **[53]** |
| --- | --- | --- | --- | --- | --- | --- | --- | --- |
| Question/objective sufficiently described? | 2 | 2 | 2 | 2 | 2 | 2 | 2 | 2 |
| Study design evident and appropriate? | 2 | 2 | 2 | 2 | 2 | 2 | 2 | 0 |
| Context for the study clear? | 2 | 2 | 2 | 2 | 2 | 2 | 2 | 1 |
| Connection to a theoretical framework/wider body of knowledge? | 1 | 2 | 2 | 1 | 1 | 1 | 1 | 0 |
| Sampling strategy described, relevant and justified? | 2 | 2 | 1 | 0 | 2 | 2 | 1 | 0 |
| Data collection methods clearly described and systematic? | 2 | 1 | 1 | 1 | 2 | 2 | 1 | 0 |
| Data analysis clearly described and systematic? | 2 | 2 | 2 | 1 | 2 | 1 | 1 | 0 |
| Use of verification procedure(s) to establish credibility? | 2 | 0 | 1 | 2 | 2 | 2 | 1 | 0 |
| Conclusions supported by the results? | 2 | 2 | 2 | 2 | 2 | 2 | 2 | 0 |
| Reflexivity of the account? | 0 | 1 | 0 | 0 | 0 | 1 | 0 | 0 |
| **Total score/possible maximum score** | **17/20** | **16/20** | **15/20** | **13/20** | **17/20** | **17/20** | **13/20** | **3/20** |

**Table 2: Quality assessment quantitative studies**

**Table 3: Assessment mixed methods studies**

| **Mixed method studies** | **[21]** | **[25]** | **[27]** | **[34]** | **[35]** | **[39]** |
| --- | --- | --- | --- | --- | --- | --- |
| **Qualitative criteria mixed methods studies** |  |  |  |  |  |  |
| Question/objective sufficiently described? | 2 | 2 | 2 | 2 | 2 | 2 |
| Study design evident and appropriate? | 2 | 2 | 2 | 2 | 2 | 2 |
| Context for the study clear? | 2 | 1 | 2 | 2 | 2 | 2 |
| Connection to a theoretical framework/wider body of knowledge? | 1 | 1 | 1 | 1 | 1 | 1 |
| Sampling strategy described, relevant and justified? | 1 | 1 | 2 | 2 | 2 | 2 |
| Data collection methods clearly described and systematic? | 1 | 1 | 2 | 1 | 1 | 2 |
| Data analysis clearly described and systematic? | 1 | 1 | 1 | 2 | 2 | 2 |
| Use of verification procedure(s) to establish credibility? | 0 | 0 | 2 | 2 | 2 | 2 |
| Conclusions supported by the results? | 2 | 2 | 2 | 2 | 2 | 2 |
| Reflexivity of the account? | 0 | 0 | 1 | 0 | 0 | 1 |
| **Quantitative criteria mixed methods studies** |  |  |  |  |  |  |
| Question/objective sufficiently described? | 2 | 2 | 2 | 2 | 2 | 2 |
| Study design evident and appropriate? | 2 | 2 | 2 | 2 | 2 | 2 |
| Method of subject/comparison group selection or source of information/input variables described and appropriate? | 1 | 1 | 2 | 2 | 2 | 2 |
| Subject (and comparison group, if applicable) characteristics sufficiently described? | 1 | 2 | 2 | N/A | 2 | 2 |
| If interventional and random allocation was possible, was it described? | N/A | N/A | N/A | N/A | N/A | N/A |
| If interventional and blinding of investigators was possible, was it reported? | N/A | N/A | N/A | N/A | N/A | N/A |
| If interventional and blinding of subjects was possible, was it reported? | N/A | N/A | N/A | N/A | N/A | N/A |
| Outcome and (if applicable) exposure measure(s) well defined and robust to measurement/misclassification bias? Means of assessment reported? | 1 | 0 | 2 | 1 | 1 | 1 |
| Sample size appropriate? | 1 | 2 | 1 | 2 | 2 | 2 |
| Analytic methods described/justified and appropriate? | 0 | 0 | 1 | 1 | 2 | 2 |
| Some estimate of variance is reported for the main results? | 1 | 1 | 2 | 2 | 0 | 2 |
| Controlled for confounding? | 0 | 0 | 1 | 1 | 2 | 0 |
| Results reported in sufficient detail? | 2 | 1 | 2 | 2 | 2 | 2 |
| Conclusions supported by the results? | 2 | 1 | 2 | 2 | 2 | 2 |
| **Total score/possible maximum score** | **25/42** | **23/42** | **36/42** | **33/40** | **35/42** | **37/42** |
