# Supplementary material for: Social media use in healthcare: A systematic review of effects on patients and on their relationship with healthcare professionals
Source: BMC Health Serv Res. 2016 Aug 26;16(1):442. doi: 10.1186/s12913-016-1691-0 (PMC5000484; doi:10.1186/s12913-016-1691-0)
Supplement: Additional file 4: — Appendix D-Summary of articles per social media category. (DOCX 14 kb) [file 12913_2016_1691_MOESM4_ESM.docx]

**Appendix D - A summary of the articles’ data source, based on social media platform and health condition**

| **Social media platform - Condition** | **Article no.** |
| --- | --- |
| Blogs - Unknown | [37] |
| Blogs - Chronic | [24] |
| Facebook/Twitter - Obesity | [30] |
| Facebook - Mental | [21, 40] |
| Facebook - Contraceptives | [17] |
| YouTube - Chronic | [16] |
| Online support community - Unknown | [1,15] |
| Online support community - Mental | [36] |
| Online support community - Chronic | [13,14, 22, 23, 25, 26, 28, 30, 35, 36] |
| Forum - Unknown | [38] |
| Forum - Mental | [34] |
| Virtual reality - Chronic | [27] |
